# Subcutaneous specific immunotherapy: Economic implications from the perspective of statutory health insurance – a population based cost-effectiveness estimation 

**DOI:** 10.5414/ALX1507E

**Published:** 2018-09-01

**Authors:** T. Reinhold, S. Willich, B. Brüggenjürgen

**Affiliations:** Institute for Social Medicine, Epidemiology and Health Economics, Charité - University Medicine, Berlin, Germany

**Keywords:** immunotherapy, allergic rhinitis, allergic asthma, cost-effectiveness

## Abstract

Background: Specific immunotherapy is the only potentially curative therapy in patients with allergic rhinitis (AR) and allergic asthma (AA). The present study examined the effects of subcutaneous immunotherapy (SCIT) on the financial situation of the German statutory health insurance systems and measures the impact on AR/AA prevalence during the next decades. A further objective was to identify possible SCIT-treatment strategies in order to reach an efficient SCIT-use. Methods: Taking population projections of the German Statistical Federal Office, the number of expected new cases (AR, AA) was calculated until 2050. Based on assumptions about the proportion of patients who received SCIT in the future, age cohorts run through a model-calculation based on Markov chains. Data on effectiveness were extracted from published literature. For determining the cost situation of SCIT pharmacies we used selling prices for Allergovit^®^. All future costs are discounted at a mean rate of 2%. The model calculation was supplemented by a Delphi panel. Results: Based on the current situation, a total annual economic burden of 540 million Euros is to be expected for care of about yearly 6 million patients with AR and AA in Germany between 2011 and 2050. Several scenarios have shown that the use of SCIT seems to be associated with cost savings from the perspective of statutory health insurances, when SCIT is offered to a larger amount of patients with moderate to severe symptoms. That would result in reduced number of expensive patients who suffer from AA. The best effects on the future number of diseased patients could be achieved, however, if SCIT additionally would be applied to patients in earlier stages of disease. Due to the large number of patients receiving SCIT in such a scenario, the initial costs would not completely compensated by cost savings. Nevertheless, the additional costs of 300 to 350 Euros per additionally healed patient seem to be justifiable. Conclusion: From the perspective of the SHI, SCIT is a useful strategic option for preventing the progression of allergic diseases. Particularly with increased use in early disease stages, the number of healed patients is high. Potential cost savings may result from increased treatment rates in patients with advanced disease stages.

**German version published in Allergologie, Vol. 35, No. 11/2012, pp. 539-550**

## Background 

Allergic Rhinitis (AR) is a frequent allergic disorder in Western societies. A study examining the prevalence rates of AR in Europe, found a self-reported frequency of 19% and a rate of 13% when diagnosis was confirmed by a physician [1]. Nevertheless, the frequency of AR seems to underlay large variations across different countries with higher rates reported particularly in Great Britain and lower rates e.g. for Spain [2]. Since the 1970s, in Western societies a continuous increase of newly diseased patients was observable, which seems to be stabilized at the moment [3]. Beside the epidemiological burden of disease, AR is further associated with a significant reduction in patients’ quality of life [4] and a substantial economic impact on health care costs and productivity. For Germany, annual mean total costs from society’s perspective of 1,089 Euro per affected child/adolescent and 1,543 Euro per adult were reported [5]. Depending on age-group, the proportion of indirect costs ranged between 22% and 58% [5]. 

Within the context of an AR-related economic burden it seems necessary to keep in mind a possible link between AR and allergic asthma (AA), because studies have suggested that AR precedes the development of an AA [3, 6]. Up to 40% of patients affected by AR may also suffer from asthma, and around 80% to 90% of patients with asthma may additionally have AR [6]. In Germany a prevalence of 3.9% was reported for AA [7]. 

So far, specific immunotherapy (SIT) is the only potentially curative therapy in patients with allergic rhinitis (AR) and was evaluated as a preventive treatment regarding the development of rhinitis related allergic asthma [8, 9]. SIT promises a symptom reduction as well as a reduced need for medication [10]. To date, a number of economic evaluations has proven SIT as a cost-effective intervention [11, 12, 13, 14, 15, 16]. Since most of these studies examined the use of SIT in a defined patient cohort, what the nationwide epidemiological as well as the economic effects of SIT are has to be investigated. 

The main objective of the present study was to assess the effects of subcutaneous immunotherapy (SCIT) on the financial situation of the German statutory health insurance system and to estimate the impact AR/AA prevalence during the next decades. Based on these findings, a further objective was to identify possible SCIT-treatment strategies in order to reach an efficient use of SCIT. 

## Methods 

### Design of the analysis 

The analysis was developed as a model-calculation based on a Markov-model approach, which reflects the course of disease of AR/AA under different treatment regimes from 2011 to the year 2050 on a population level. 

The decision to conduct such a model approach was made in order to synthesize data from multiple sources. In cases where no valid data sources were available, we made use of expert judgements. For this purpose, we performed a concomitant expert board composed of allergy experts in pediatrics and otolaryngology. 

### Economic perspective and time-adjustment 

The analysis was made from the perspective of the statutory health insurance systems in Germany. Thus the analysis does not take into account indirect costs (e.g. due to disease related absence from work, early retirement etc.). 

Since the analysis covers a projected time horizon up to year 2050, any future costs were discounted using a mean annual discounting rate of 2%. 

The model calculation was performed using MS Excel 2007. 

### Description of the analysis 

**Figure 1.**
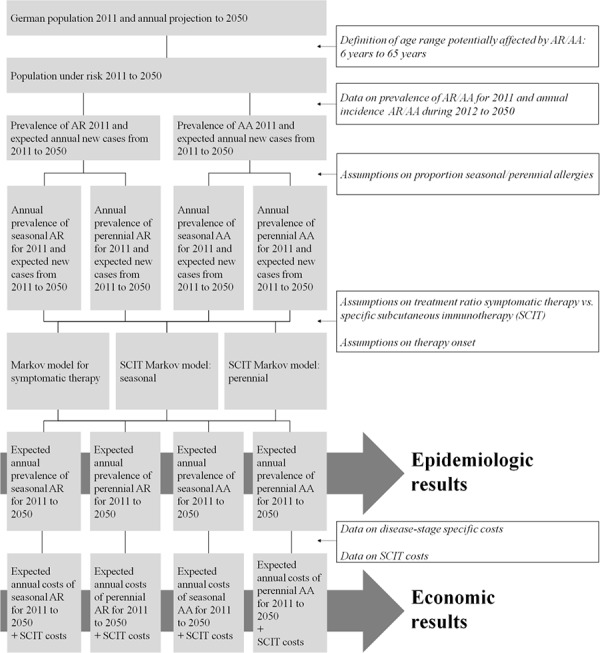
The budget impact model description – basic operating principles (Abbreviations: AA – Allergic Rhinitis, AR – Allergic Asthma, SCIT – Subcutaneous immunotherapy).

The model calculation consists of different modules. These modules and the basic operating principles are illustrated in Figure 1. 

First it was necessary to get information on the structure of the German population for 2011 and annual-based population projection until 2050. These data were provided by the German Federal Statistical Office [17]. Data include a gender-specific breakdown of the German population divided by age. We supposed AR or AA is negligible in younger age groups as well as in elderly. Based on expert’s estimations, the population under risk was defined as the German population aged between 6 and 65 years for each year between 2011 and 2050. To determine the number of patients who were already affected by AR and/or AA at the beginning of the analysis we used current prevalence data for Germany [2, 7]. Thereby we distinguished between children (6 to 12 years), adolescents (13 to 18 years) and adults (19 to 65 years). We further assumed a mean relative frequency of 60% for seasonal and 40% for perennial types of disease [18]. 

The central element of the present investigation was the use of Markov-models (Markov-chains) to portray the long-term course of AR or AA. Markov-models are appropriate for modelling of diseases that progress over time [19]. This approach has been chosen as either directly after the manifestation of AR or during the course of the disease both a worsening (e.g. development of serious AR or AA), or an improvement (such as spontaneous healing), were possible [20, 21, 22]. 

**Figure 2.**
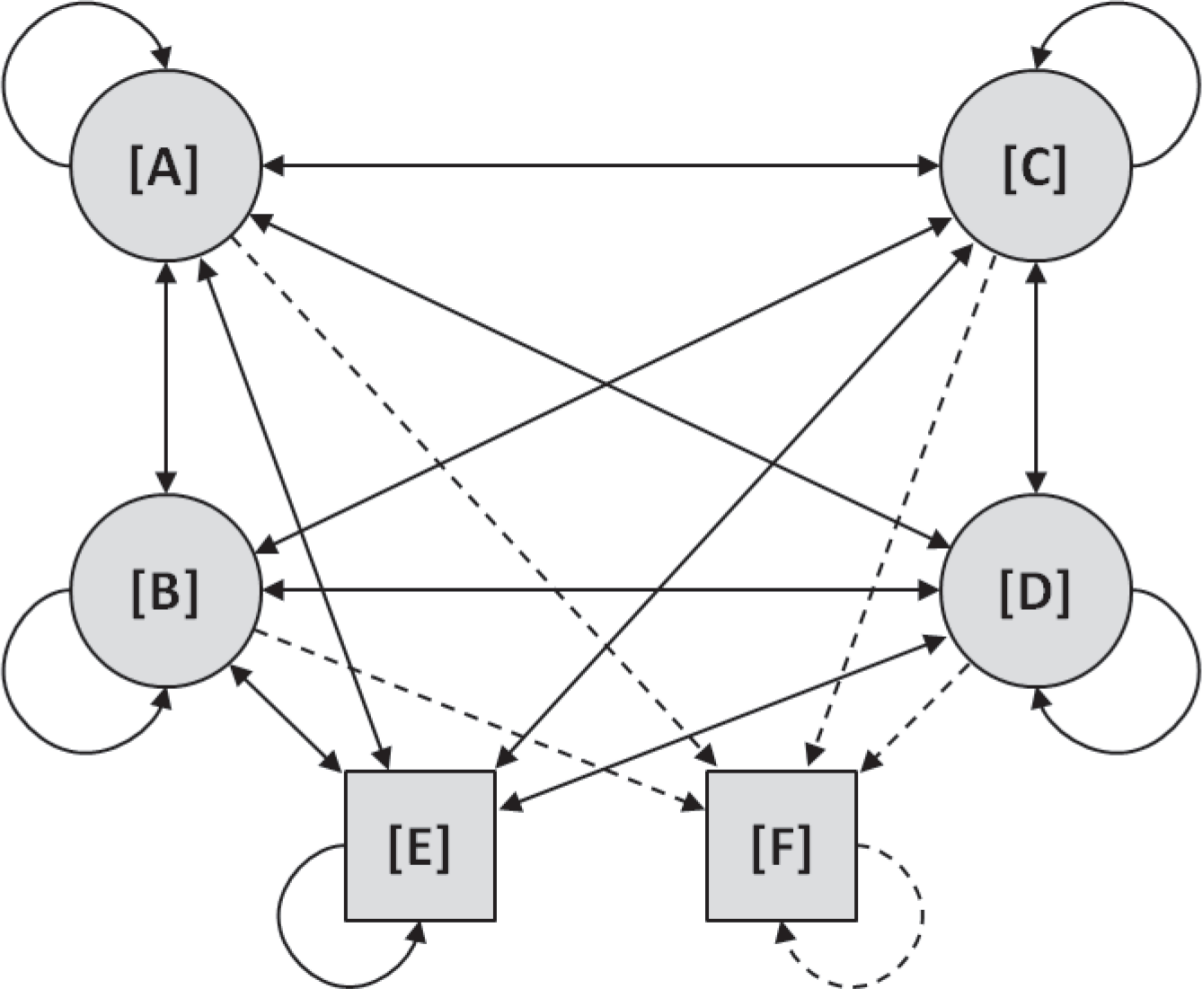
Markov-model structure with disease-states and conditions (Abbreviations: [A] – mild AR, [B] – moderate/severe AR, [C] – moderate/severe AR + mild AA, [D] – severe AR + moderate/severe AA, [E] – no symptoms/healthy, [F] – death).

Therefore, the currently diseased patients as well as the expected new cases pass these Markov-models according to scenario-specific assumptions on the proportion of patients receiving SCIT. Different Markov-models were developed for children, adolescents and adults depending on the type of allergy (seasonal, perennial) and the kind of treatment (SCIT, symptomatic therapy (ST)). Each model consists of four predefined disease states [A] to [D] ([A] *mild AR*, [B] *moderate/severe AR*, [C] *moderate/severe AR + mild AA*, [D] *severe AR + moderate/severe AA*) as well as the states [E] *No symptoms/healthy* and [F] *death. *The length of each Markov-cycle was defined to be one year. All events or progression are represented as transitions from one state to another with a certain probability. The transition probabilities between pre-defined states were obtained either directly or derived from published literature sources [11, 20, 21, 23, 23, 25, 26, 27, 28, 29]. The principle structure of the underlying Markov-models are illustrated in Figure 2. Further details of underlying Markov-models are already described elsewhere [12]. Using these Markov-models the expected number of patients in each health state was measurable for each year of the projection. 

Spending one cycle in a given state is thereby associated with certain costs. These health-state specific costs were calculated based on a large German pilot project, in which also patients with different allergic diseases were examined [30]. By summing up the cycle-specific cost values ​​over the total time horizon of the analysis, the expected economic impact was determinable. 

Additionally, the costs for SCIT were taken into account. For determining the cost situation of SCIT products, we used an average of pharmacy selling prices of perennial and preseasonal use of Allergovit^®^. Independently from the kind of application, mean costs of 382 Euro were considered for the first year of SCIT-application, followed by mean costs of 371 Euro during year two to three after SCIT onset. These mean values are resulting from the assumption, that for pre-seasonal SCIT-application annual costs of 262 Euro occur for three years, while for perennial application costs of 502 Euro in year one, and 480 Euro in year two and three were considered, respectively. Costs for outpatient visits associated with SCIT-treatment were not involved, due to the fact, that outpatient physician services in Germany are paid by a fixed budget per insured patient to the physicians association, regardless of whether an insured person will really contact his physician or not. Thus, from the perspective of a health insurance company it doesn’t matter how many outpatient contacts an insuree experienced. If the number of contacts will increase, the per-contact-payment rate of a German physician will decrease at the same time. 

Since one of the main objectives of the study was to measure the impact of SCIT on the total financial situation of the German health insurance systems, different scenarios regarding the SCIT-treatment were defined. These scenarios differ regarding the proportion of patients who receive SCIT and the disease state in which the SCIT will start to be administered. Based on results of our expert panel it was assumed, that SCIT in year 2011 is available for 5% of seasonal affected children, 15% of adolescents and 20% of adults (perennial affected patients: 5% of children, 10% of adolescents and 20% of adults). The following scenarios were considered: 

Status Quo-scenario: According to the expert assumptions, the proportion of patients receiving SCIT will increase to 10% of seasonal affected children, 20% of adolescents and 30% of adults until year 2030 (perennial affected patients: 5% of children, 15% of adolescents and 30% of adults). The model assumes a linear change of SCIT supply rates from 2011 over time. This status quo scenario implies that SCIT will be administered primarily in patients suffering from advanced states of disease (Therapy onset in diseases states [C] and [D]).

- Scenario 1: The SCIT supply rates of 2011 will triple already until 2020 in all age groups. Comparable to Status Quo-scenario it is assumed, that SCIT will be administered primarily in patients suffering from advanced states of disease (Therapy onset in diseases states [C] and [D]).
- Scenario 2: The SCIT supply rates will reach 75% until 2020 in all age groups. Comparable to a status quo-scenario it is assumed, that SCIT will be administered primarily in patients suffering from advanced states of the disease (Therapy onset in diseases states [C] and [D]).
- Scenario 3: The increase in SCIT supply rates reach the same level as was described for the status quo-scenario. The main difference is that SCIT was additionally offered to patients already in earlier diseases states (Therapy onset in diseases states [B], [C] and [D]).
- Scenario 4: The SCIT supply rates for 2030 were the same as in the status quo-scenario, but it was assumed that SCIT will be offered to patients in all diseases-states (Therapy onset in diseases states [A], [B], [C] and [D]).

All analyses were made with regard to possible uncertainties particularly due to the number of assumptions and unproven expert opinions. Therefore, the calculation was performed as a probabilistic analysis to proof the results for robustness. All scenarios were recalculated 1,000 times under randomly varied input parameters in order to get information on scatter of results and to give a statistical measure of dispersion in terms of standard derivation. All important variables used in the model calculation as well as data range considered in our analyses are mentioned in Table 1. 

To get information on cost-effectiveness we had a further look on differences in effects between all investigated scenarios. In cases of superior effects compared to status quo-scenario, we calculated the incremental cost-effectiveness ratio, showing the costs per additional healed resp. avoided patient. 

## Results 

### Epidemiology

In 2011 the number of patients was expected to be about 7.4 ± 0.7 million Germans. Driven by the demographic development in Germany and assuming a stable incidence of allergic diseases, the absolute number of affected patients is expected to decrease during the coming decades. 

**Figure 3.**
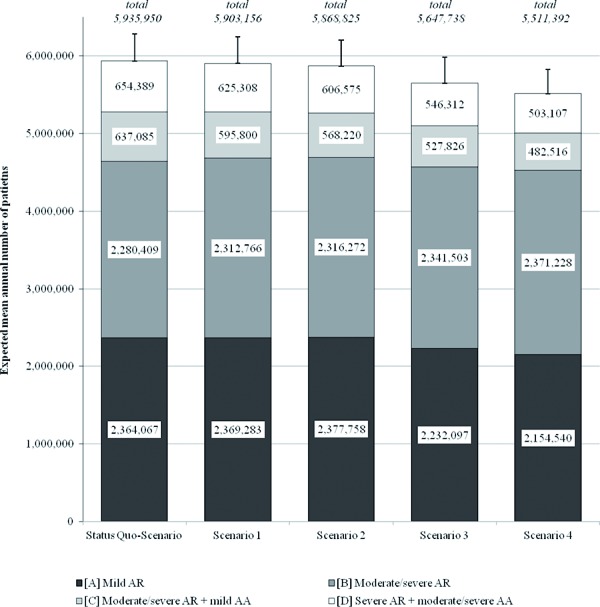
Expected annual mean number of patients (Abbreviations: AA – Allergic Rhinitis, AR – Allergic Asthma).

Over the total time period from 2011 to 2050, the expected number of patients would be the highest in the status quo-scenario (Figure 3). So, the mean annual number of patients would be 5.9 ± 0.4 million. If SCIT would be offered to a larger amount of moderate to severe affected patients (see scenario 1, 2) the patient numbers could be reduced moderately. A larger positive effect on the absolute number of patients would be realizable if SCIT use would extended also to mild affected patients in earlier disease stages (scenario 3, 4). A reduction of patient numbers is particularly detectable in more severe disease stages. Compared to status quo-scenario, the expected annual number of patients suffering from AA (disease state C, D) might be reduced from 5% in scenario 1 up to 24% in scenario 4. 

### Economic

**Figure 4.**
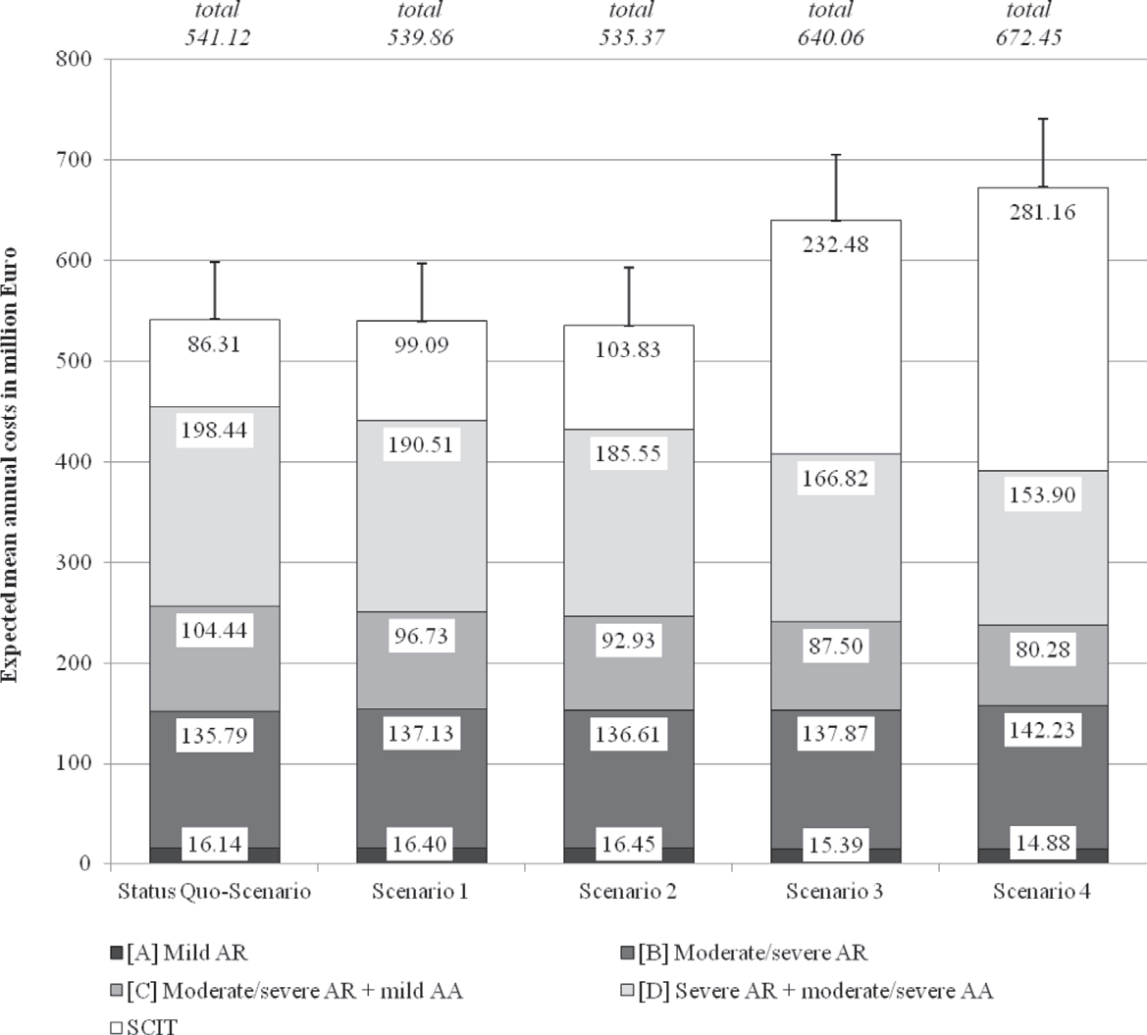
Expected annual mean costs (Abbreviations: AA – Allergic Rhinitis, AR – Allergic Asthma, SCIT – Subcutaneous immunotherapy).

From the point of view of statutory health insurance companies in Germany, the status quo-scenario is associated with mean annual total costs of 541.1 ± 57.7 million Euro, while 86.3 ± 11.0 million Euro are directly apportionable to SCIT (see Figure 4). If SCIT would be offered to more severely diseased patients (scenario 1, 2) direct mean cost savings of annual 1.3 million (scenario 1) to 5.7 million Euro (scenario 2) could result. The initial higher costs for SCIT would be compensated by savings due to less severe affected patients in the future. 

Other findings result for scenarios in which SCIT would additionally be offered to patients in milder disease stages, such as mild AR. Although an extension of SCIT to marginal affected patients (scenario 3, 4) would reach a substantial number of healed resp. improved patients, such a therapeutic approach would also lead to significant higher costs over the investigated time period. The main explanation for this finding is the fact that most patients suffer from mild stages, so the number of additional patients treated with SCIT would strongly increase as well as the related SCIT-costs. On the other hand, the cost-saving potential is comparably low, especially due to low mean costs that are related with mild disease stages. 

### Cost-effectiveness-measurement

Since a primary aim of any health economic research is to optimize the value of investments in national healthcare systems, we were further interested to calculate the cost-effectiveness level of the investigated scenarios. 

In comparison to status quo, any increase in SCIT-supply rates results in less patients suffering from allergic disorders. Taking into account the overall cost results, scenario 1 and 2 are clearly cost-effective, because the overall costs are lower from the perspective of German statutory health insurance and the scenarios reach a superior effectiveness. 

Superior effects on the future number of diseased patients could be achieved, however, if SCIT would already be applied to patients in earlier states of disease. Due to the large number of patients receiving SCIT in such a scenario, the initial costs would not completely be compensated by cost savings. Nevertheless, the additional costs of 300 to 350 Euros per additionally healed patient seem to be justifiable (see Table 2). 

## Discussion 

The present investigation indicates SCIT as a treatment option for allergic patients and offers a useful way to influence the burden of disease. Depending on the way SCIT is administered to the patients, this kind of treatment might be able to attain an economic benefit from the view of statutory health insurance systems. Particularly when SCIT would be offered to a larger amount of moderate to severe diseased patients, a cost-saving potential exists. Of course, the increase in treatment rates in these patient clientele natural bounds, as for example the presence of a severe asthma presents as a contraindication for SCIT according to current recommendations [31]. Otherwise SCIT is associated with a larger potential for reducing the number of patients when adopted in already mild affected patients. The resulting additional costs of 300 to 350 Euros per cured patient gained seem to be justifiable. 

So far, there is a lack of comparable studies on allergic treatment strategies, which focused on cost-effectiveness in terms of costs per cured patient. With regard to respiratory disorders we found just one Dutch study investigating the cost-effectiveness of different therapies for managing acute sinusitis. Compared to strategy “wait and see”, the authors found the costs for curing one additional patient were 230 Euro when antibiotics were selectively prescribed, and 400 Euro when antibiotics were immediately prescribed [32]. 

To our knowledge this is the first investigation assessing the use of SCIT on a population-based level. Until now, most studies are investigating a predefined patient cohort. In such studies, the assessment of cost-effectiveness is usually improved compared to population-based approaches. This is mainly due to the fact that every year new diseased patients, requiring treatment too, have to be considered in population-based studies. Thus, costs for SCIT occur during the total study period, and not just at the beginning of the investigation. 

Another factor influencing the economic assessment of the study is caused by the long-term view of the present investigation. Due to the adjusting for differential timing of costs by using a mean discounting-rate of 2% per annum, the future cost savings were valued lower when viewed from the present. Although the process of discounting is basically accepted in health economic research [33] it is consistently a subject of discussion particularly for long-term health care programs where benefits mainly appear in the future [34]. Discounting potentially makes nearly all prevention programs aimed at long term benefits more cost-ineffective. This also counts for SCIT in a long-term view. Considering this background, the present model could be seen as a more conservative approach. 

Another subject for discussion could be seen with regard to the expected number of patients during the coming decades. Different studies exist which tried to give measurements regarding the prevalence of AR and AA in the future. Some studies suggest an increase in relative patient numbers [35, 36], other investigations assume a stable frequency [37, 38, 39]. For the present analysis the underlying Markov-models assume in its incidence rates, that the relative change of affected patients between 2011 and 2050 would be the same as the change in Germany’s total population under risk during the same time if no SCIT would be offered. A future increase of prevalence rates would also influence the cost-effectiveness assessment of SCIT. Particularly a larger number of severe diseased patients can result in an increased cost-saving potential of SCIT. Also changes in selling prices for SCIT products have a direct effect to SCITs cost-effectiveness. When assuming a decrease in such prices during the future, e.g. after expiration of patent protections or due to increased competition between pharmaceutical companies, the cost-effectiveness of SCIT would be improved too. 

Last but not least the cost data used for different predefined Markov-states could be discussed. Currently there are no valid published data available, which describe the economic burden associated with allergic disease states in Germany from the view of statutory health insurances. For that reason we have used own data extracted and calculated based on another study project [30]. Assuming that real costs may be higher than these we have used in our model calculation, then they would also have positive effects regarding cost-effectiveness of SCIT. Especially disease specific annual cost data of disease state [A] (mild AR) of our model seem comparably low (8 to 27 Euro). But in the absence of more valid cost data we had to use these data. Moreover, it is to assume that a lot of costs are paid by patients themselves. If these costs would be considered additionally, the cost-effectiveness assessment of SCIT would probably be affected positively. 

## Conclusion

From the perspective of the statutory health insurance systems in Germany, SCIT is a useful strategic option for preventing the progression of allergic diseases. Particularly with increased use in early disease stages, the number of healed patients is high. Potential cost savings may result from increased treatment rates in patients with advanced disease stages. 

## Conflict of interest

The study was financially supported by Allergopharma Joachim Ganzer KG, Germany. The design and implementation of the analysis was solely the responsibility of the authors. 

**Table 1.** Important input variables used in the analysis (Abbreviations: AA – Allergic Rhinitis, AR – Allergic Asthma, SCIT – Subcutaneous immunotherapy, [A] – mild AR, [B] – moderate/severe AR, [C] - moderate/severe AR + mild AA, [D] – severe AR + moderate/severe AA, [E] – no symptoms/healthy, [F] – death).

Input item	Values used in the model calculation (mean [range used for probabilistic analyses])
Population under Risk	6 to 65 years
Prevalence AR	Children: 4.7% [3.00% to 7.00%] [2] Adolescents: 16.9% [10.00% to 20.00%] [2] Adults: 17.95% [15.00% to 20.00%] [7]
Prevalence AA	Children: 3.6% [2.00% to 5.00%] [2] Adolescents: 5.7% [4.00% to 7.00%] [2] Adults: 3.9% [2.00% to 5.00%] [7]
Proportion of seasonal affected cases	60% [45% to 75%] [18]
Annual costs from third party payers perspective according to diasese-state	*Children* [A] mild AR: 27 Euro [19 to 35 Euro] [B] moderate/severe AR: 119 Euro [83 to 155 Euro] [C] moderate/severe AR + mild AA: 282 Euro [197 to 367 Euro] [D] severe AR + moderate AA: 446 Euro [312 to 580 Euro] *Adolescents* [A] mild AR: 18 Euro [13 to 23 Euro] [B] moderate/severe AR: 97 Euro [68 to 126 Euro] [C] moderate/severe AR + mild AA: 259 Euro [181 to 337 Euro] [D] severe AR + moderate AA: 442 Euro [309 to 575 Euro] *Adults* [A] mild AR: 8 Euro [6 to 10 Euro] [B] moderate/severe AR: 79 Euro [55 to 103 Euro] [C] moderate/severe AR + mild AA: 227 Euro [159 to 295 Euro] [D] severe AR + moderate AA: 442 Euro [309 to 575 Euro] source: own calculation [30]
Immunotherapy costs	Year 1: 382 Euro [262 to 502 Euro] Year 2: 371 Euro [262 to 480 Euro] Year 3: 371 Euro [262 to 480 Euro] source: Pharmaceutical Manufacturer Information
Discount rate	2.00% p.a. [1.5% p.a. to 2.5% p.a.]

**Table 2.** Differences in the expected population-based annual cost and the annual number of cured patients compared to status quo-scenario.

**Scenario **	**Additional mean annual costs **	**Additional mean annual healed/avoided patients **	**Costs per additionally healed patient**
1	– 1,250,059 Euro	32,794	– 38
2	– 5,732,459 Euro	67,126	– 85
3	98,964,793 Euro	288,213	343
4	131,341,777 Euro	424,559	309
